# Short-term poor glycemic control and retinal microvascular changes in pediatric Type 1 Diabetes patients in Singapore: a pilot study

**DOI:** 10.1186/s12886-017-0449-8

**Published:** 2017-06-15

**Authors:** Ling-Jun Li, Ecosse Lamoureux, Tien Yin Wong, Ngee Lek

**Affiliations:** 1 Singapore Eye Research Institute, Singapore National Eye Centre, Singapore, Singapore; 2 Duke-NUS Medical School, Department of Pediatrics, Singapore, Singapore; 3 Department of Pediatrics, KK Women’s and Children’s Hospital, Singapore, Singapore

**Keywords:** Glycemic control, Retinal microvascular changes, Type 1 Diabetes (T1D), Children

## Abstract

**Background:**

Poor glycemic control in Type 1 Diabetes (T1D) patients is strongly associated with an increased risk of diabetes-related microvascular complications later in life, but it is unclear whether short period of poor glycemic control in children with T1D can cause evident microvascular morphological changes long before any pathological manifestation. Our study aimed to investigate the longitudinal association between poor glycemic control and subsequent changes in retinal microvasculature, in a pilot study of 55 pediatric T1D patients from Singapore after a one-year follow-up.

**Methods:**

This is a hospital-based, exposure-matched and retrospective longitudinal study. A total of 55 T1D patients were included from Singapore KK Women’s and Children Hospital, 28 of whom had poor glycemic control (average glycated hemoglobin [HbA1c] ≥8% during the year) while the other 27 age- and gender-matched subjects had good glycemic control (HbA1c <8%). Retinal photography was taken at diabetes annual screening and images were graded by trained graders using a semi-automated computer-based program (Singapore I Vessel Assessment [SIVA], version 4.0, Singapore Eye Research Institute, Singapore) and a spectrum of retinal vascular parameters (e.g. caliber, tortuosity, branching angle and fractal dimension) were measured quantitatively from 0.5 to 2.0 disc diameters.

**Results:**

There was no significant difference in ethnicity, duration of T1D, blood pressure, body mass index (BMI) and low-density cholesterol lipoprotein (LDL) between the two groups. Retinal imaging was obtained at the end of 1 year of glycemic control assessment. In multiple linear regression adjusting for ethnicity, BMI, LDL and duration of T1D, patients with poor glycemic control tended to have marginally wider retinal arteriolar caliber (6.0 μm, 95% CI: −0.9, 12.8) and had significantly larger retinal arteriolar branching angle (10.1 degrees, 95% CI: 1.4, 18.9) compared with their age- and gender- matched counterparts with good glycemic control.

**Conclusions:**

Our findings showed that abnormal retinal microvascular morphology was evident in pediatric patients with T1D after one-year’s poor glycemic control. Such morphological abnormalities may lead to future development of microvascular complications among T1D pediatric patients with poor glycemic control.

## Background

Type 1 Diabetes (T1D) is a chronic disease characterized by elevated blood glucose which results from lack of endogenous insulin subsequent to autoimmune destruction of pancreatic beta cells [1]. Hyperglycemia is known to be associated with increased shear stress and impaired microvascular endothelium [2] which leads to microvascular complications such as retinopathy, neuropathy and nephropathy [3]. Following diagnosis of T1D in young subjects, optimal glycemic control is strongly recommended as it is associated with a reduced risk of diabetes-related complications later in life [4].

Substantial evidence has shown that retinal imaging is a non-invasive, reproducible and reliable technology to view small vessel morphology, and changes in these microvascular features can reflect the degree of endothelial dysfunction in T1D patients [3]. In several cross-sectional population-based studies, retinal arteriolar and venular widening, increased retinal arteriolar tortuosity and enlarged retinal arteriolar branching angle were associated with increased concentrations of fasting plasma glucose (FPG) and glycated haemoglobin (HbA1C), as well as a longer duration of T1D both in children and adults [5, 6]. However, it is unclear whether such changes are already evident after a short period of poor diabetes control in children with T1D.

As poor glycemic control may cause endothelial dysfunction in vivo*,* and can be reflected in real-time retinal microvascular changes, we investigated the longitudinal association between glycemic control and subsequent changes in retinal microvasculature, in a pilot study of 55 pediatric T1D patients from Singapore after a one-year follow-up. We hypothesized that short-term poor glycemic control is associated with suboptimal retinal vascular morphology in pediatric patients with T1D.

## Methods

### Study design

This is a hospital-based, exposure matched and retrospective longitudinal study. Twenty-eight pediatric T1D patients aged 10–16 years with poor glycemic control and 27 age- and gender- matched pediatric T1D patients with good glycemic control who attended follow-up outpatient appointments in the diabetes clinical service at KK Women’s and Children’s hospital (KKH) in Singapore, were included in the present analyses. All the patients were on insulin replacement treatment and were not complicated by any degree of diabetic retinopathy (DR). Eligible subjects were Singapore citizens or long-term residents, who had quarterly HbA1C measurements and annual retinal screening test according to the American Diabetes Association (ADA) 2016 guidelines (http://www.ndei.org/ADA-diabetes-management-guidelines-microvascular-complications-neuropathy-retinopathy-nephropathy-diabetic-kidney-diseasefoot-care.aspx.html). This study was approved by SingHealth Centralized Institutional Review Board, and was conducted according to the tenets of the Declaration of Helsinki.

### Exposure—Glycemic control

Venous whole blood specimens were collected in EDTA tubes at each 3-monthly clinic visit. HbA1C was measured using the Cobas Integra 800 (Roche Diagnostics, Rotkreuz, Switzerland), based on the turbidimetric inhibition immunoassay for hemolyzed whole blood (reference range 4.4–6.4%). HbA1C measurements were standardized to the reference method aligned with the Diabetes Control and Complications Trial and the American National Glycohemoglobin Standardization Program standards [7]. The intra-assay coefficient of variation was 2.3% and the inter-assay coefficient of variation was 2.4%. Since venous whole blood specimens were collected in EDTA tubes at a quarterly clinic visit, we calculated average HbA1C at a yearly basis with four readings. For the purpose of the present analyses, good glycemic control was defined as an average HbA1C value of less than 8% within a year, while poor glycemic control was defined as 8% and above [8].

### Outcome—Retinal microvascular assessment

Retinal photography was performed at the year-end clinic visit. Right eye digital retinal photographs were taken from participants using a 45° non-mydriatic retinal camera (Canon CR-1, 40D SLR digital retinal camera backing; Canon Inc., Japan) after pharmacological pupil dilation. Two retinal images centered on the optic disc and macular were taken. Retinal photographs were assessed by trained graders using a semi-automated computer-based program (Singapore I Vessel Assessment [SIVA], version 4.0, Singapore Eye Research Institute, Singapore) and a spectrum of retinal vascular parameters were measured quantitatively from 0.5 to 2.0 disc diameters (zone C) away from the optic disc margin. [9] Retinal vascular caliber, branching angle, tortuosity and fractal dimension were assessed according to standard protocol, which have been described in detail in previous publications [9]. Intra-grader reliability was assessed in 10% (*n* = 6) of randomly selected retinal photographs from our study. The intra-class correlation coefficient (ICC) was above 0.90 for all retinal vascular parameters.

### Co-variates

Data on duration of T1D, ethnicity, parental education and housing condition were collected at the baseline visit. Patients’ weight, height, systolic (SBP) and diastolic (DBP) blood pressure were measured at baseline visit, and type of T1D medication, pubertal stage, foot review and dietician review were evaluated at diabetic screening clinic at each follow-up visit. Body mass index (BMI) was calculated as weight (kg) divided by square of height (m^2^). Fasting glucose, cholesterol, high-density cholesterol lipid (HDL), low-density cholesterol lipid (LDL), triglyceride, free thyroxine (T4), thyroid stimulating hormone and urine albumin/creatinine ratio (uACR) were examined at each visit.

### Statistical analysis

Glycemic control (good vs. poor) was analysed as a dichotomous variable. Retinal microvascular measures were normally distributed and analysed as continuous variables. Comparisons of baseline variables were examined using a 2-sample independent student t-test (for continuous variables), χ^2^ test or fisher exact t-test (for categorical variables).

Multiple linear regression was used to analyse the association between one-year glycemic control (poor vs. good) and retinal microvascular measures. Since this is an exposure-matched cohort, four models were tested: Model 1-unadjusted; Model 2-adjusted for ethnicity; Model 3-Model 2 and additionally adjusted for baseline BMI and LDL; Model 4-Model 3 and additionally adjusted for duration of T1D. Pubertal stage and uACR were also included into the fully-adjusted model in a sensitivity analysis. Mean difference in estimates and 95% confidence interval were described. All regression analyses were performed using SPSS 19.0 (SPSS Inc., Chicago, U.S.). Potential effect modifications by ethnicity and child sex were examined by including multiplicative terms with glycemic control (HbA1C*ethnicity, HbA1C*child sex) into the fully-adjusted model.

## Results

Comparisons of baseline variables between subjects with good (*n* = 27) and poor glycemic control (*n* = 28) are described in Table 1. No significant differences were found between both groups in terms of age, gender, ethnicity, duration of T1D, SBP, DBP, BMI, LDL and uACR at baseline. Compared to subjects with good glycemic control, those with poor glycemic control had wider retinal arteriolar caliber (139.84 vs. 133.81 μm, *p* = 0.03) and larger retinal arteriolar branching angles (85.76 vs. 78.28 degree, *p* = 0.04).

**Table 1:** Comparisons of clinical characteristics between T1D pediatric patients with good and poor glycemic control

	Good glycemic control	Poor glycemic control	*p* value*
	(*n* = 27)	(*n* = 28)	
	mean, SD or n, %	mean, SD or n, %	
Baseline characteristics,			
Age, years	13.3, 1.9	13.0, 1.8	0.45
Sex, male	14, 51.9%	11, 39.3%	0.35
Ethnicity,			
Chinese	20, 74.1%	13, 46.4%	0.05
Malay	2, 7.4%	9, 32.1%	
Indian	5, 18.5%	6, 21.4%	
Duration of diagnosis, years	4.17, 3.8	5.64, 3.1	0.12
Anthropometric measures,			
SBP, mmHg	106.2, 10.8	104.6, 11.2	0.85
DBP, mmHg	64.9, 7.1	61.7 5.8	0.10
BMI, kg/m^2^	19.4, 3.2	20.4, 2.9	0.25
Serum biomarkers,			
LDL, mmol/L	2.9, 0.9	3.1, 0.9	0.49
uACR, mg/g	1.4, 1.8	1.0, 0.8	0.60
Retinal vascular parameters			
Retinal arterioles,			
Caliber, μm	133.8, 10.6	139.8, 9.5	*0.03*
Fractal dimension, Df	1.22, 0.05	1.21, 0.06	0.24
Branching angle, D	78.3, 14.7	85.8, 11.8	*0.04*
Curvature Tortuosity, unit	9.1E-5, 1.7E-5	9.8E-5, 2.5E-5	0.27
Retinal venules,			
Caliber, μm	188.2, 13.1	196.3, 22.3	0.11
Fractal dimension, Df	1.22, 0.04	1.20, 0.04	0.11
Branching angle, D	77.7, 10.9	77.1, 9.8	0.85
Curvature Tortuosity, unit	9.4E-5, 1.7E-5	9.5E-5, 1.5E-5	0.81

*student t-test, χ^2^ test or fisher exact test.

*Abbreviation*: *SD* standard deviation, *SBP* systolic blood pressure, *DBP* diastolic blood pressure, *BMI* body mass index, *LDL* low-density cholesterol lipoprotein, *uACR* Urine albumin to creatinine ratio

After adjusting for ethnicity, baseline BMI and LDL and duration of T1D, pediatric patients with one-year poor glycemic control tended to have marginally wider retinal arteriolar caliber (6.0 μm, 95% CI: −0.9, 12.8) and significantly larger retinal arteriolar branching angle (10.1 degree, 95% CI: 1.4, 18.9), compared with their age- and gender- matched counterparts with good glycemic control (Table 2). Retinal arteriolar tortuosity, fractal dimension and all venular parameters however, were not associated with poor glycemic control. No potential effect modifications by ethnicity or child sex were observed.

**Table 2:** Association between poor glycemic control and retinal microvascular parameters in T1D pediatric patients

	Retinal arteriolar caliber, μm Difference in β (95% CI)	Retinal arteriolar branching angle, degree Difference in β (95% CI)
Good glycemic control	Reference	Reference
Poor glycemic control		
Model 1	*6.0 (0.6, 11.5)*	*7.5 (0.3, 14.7)*
Model 2	5.3 (−0.5, 11.1)	*8.0 (0.4, 15.6)*
Model 3	6.3 (−0.3, 12.9)	*9.1 (0.5, 17.7)*
Model 4	6.0 (−0.9, 12.8)	*10.1 (1.4, 18.9)*

Model 1, unadjusted.

Model 2, adjusted for ethnicity.

Model 3, Model 2 and additionally adjusted for BMI and LDL at baseline.

Model 4, Model 3 and additionally adjusted for duration of T1D diagnosis.

**Fig. 1:**
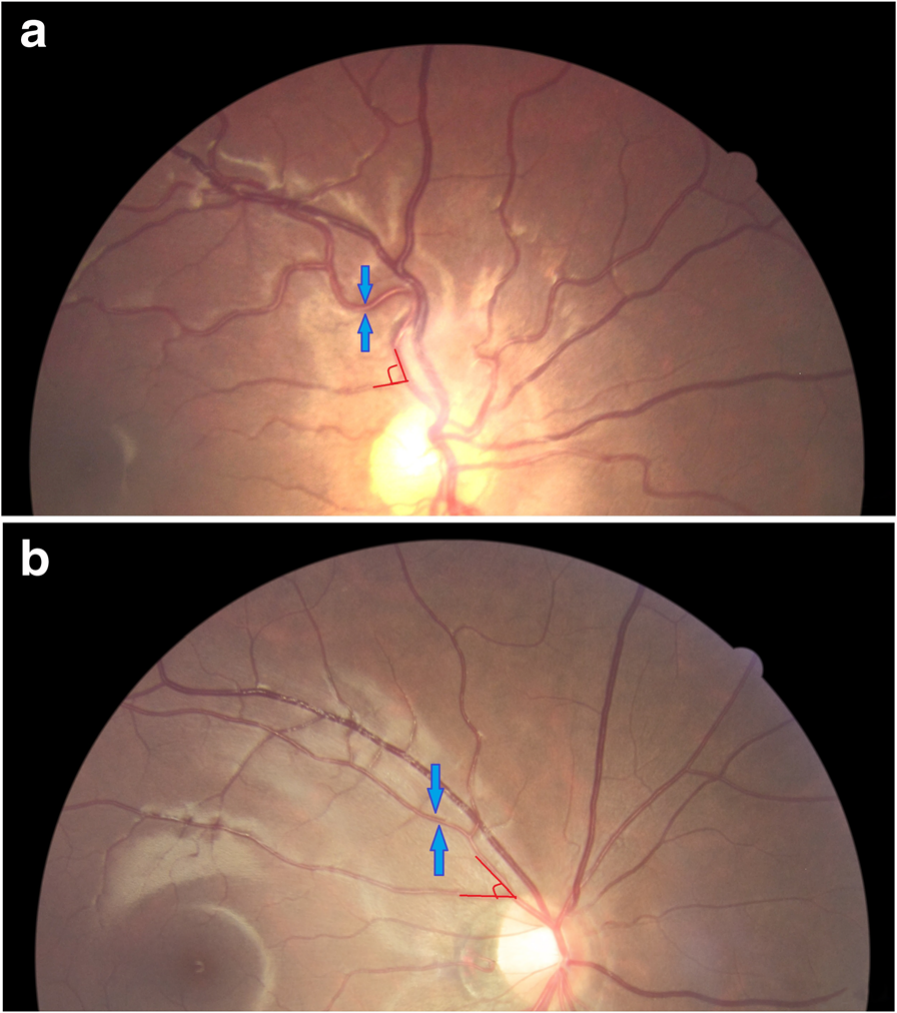
Retinal images of a T1D patient with poor glycemic control and a T1D patient with good glycemic control. Comparison of retinal arteriolar caliber and retinal arteriolar branching angle between patient with poor glycemic control (**a**) and patient with good glycemic control (**b**). *Blue arrows* are pointing to the width of retinal arterioles while red angles are indicating retinal arteriolar branching angle. The patient with poor glycemic control had a wider average retinal arteriolar caliber (147.8 vs. 112.3 μm) and a larger average retinal arteriolar branching angle (110.4 vs. 90.0°) than the patient with good glycemic control

Figure 1 showed the difference in retinal arteriolar caliber and retinal arteriolar branching angle between T1D patients, one of which had 1-year good glycemic control while the other had one-year poor glycemic control.

## Discussion

In this pilot study, we found that short-term poor glycemic control was associated with wider retinal arteriolar caliber and larger retinal arteriolar branching angle in a sample of pediatric T1D patients in Singapore.

Owing to the non-invasive and reproducible nature of the retinal microvasculature imaging, changes in retinal vascular geometry have been extensively studied and validated as proxies for small-vessel dysfunction, particularly in diabetes and other microvascular disorders [3]. We found that short-term poor glycemic control was associated with suboptimal retinal microvascular morphology including wider retinal arteriolar caliber and larger retinal arteriolar branching angle. Small-vessel dysfunction has been shown to be a consequence of T1D, possibly as a result of endothelial dysfunction and inflammation resulting from chronic hyperglycemia [10, 11]. Increased vascular shear stress and impaired microvascular endothelium mediated by hyperglycemia has been demonstrated in diabetic patients, and chronic hyperglycemia has been proven to lead to vasodilation of retinal arterioles and venules [12, 13]. Furthermore, a larger branching angle would increase the workload and energy spent, and thereby reducing the efficiency of retinal blood flow [14]. Such alterations in branching angle have been correlated with atherosclerosis, deranged blood flow and endothelial damage [15]. In our study, we found that both wider caliber and enlarged branching angle in retinal arterioles was associated with higher HbA1C episodes in the past year. Such morphological abnormalities may indicate a worse hypoxia-induced formation and a lower complexity of less than ideal oxygenation caused by chronic hyperglycemia, which may shed light on future development of microvascular complications (i.e. retinopathy) among such poor glycemic control pediatric patients.

In this short-term longitudinal study, measurements for both exposure and outcome followed standardized protocols and were analyzed by qualified staff. This study however, is not without limitations. First, our study had a relatively small sample for analysis, which might have potential selection bias in truly representing the pediatric T1D population in Singapore. Second, the small sample size might not be sufficiently powered to detect other retinal vascular parameters in relation to poor glycemic control. Third, residual confounding (i.e. compliance to insulin treatment) due to unmeasured/uncollected confounders might potentially affect our analysis. Future studies with a larger sample size and a longer follow-up are warrant.

In conclusion, our findings showed that poor glycemic control in the past year was associated with morphological abnormalities in retinal microvasculature (wider retinal arteriolar caliber and enlarged retinal arteriolar branching angle) in a sample of Singapore T1D pediatric patients aged 10–16 years. These morphological abnormalities may indicate a worse small-vessel function caused by poor glycemic control, which may lead to future development of microvascular complications among T1D pediatric patients with suboptimal glycemic control.

## Conclusions

In our hospital-based, exposure-matched and retrospective longitudinal study, a total of 55 pediatric T1D patients were included for analysis after a year’s insulin treatment. Our findings showed that abnormal retinal microvascular morphology was evident in pediatric T1D patients with short-term poor glycemic control. Therefore, we suggested that morphological abnormalities of retinal microvasculature (i.e. arteriolar widening and arteriolar branching angle enlargement) may lead to future development of microvascular complications among T1D pediatric patients with poor glycemic control.

## Abbreviations

T1D: Type 1 diabetes
FPG: Fasting plasma glucose
HbA1C: Glycated haemoglobin
DR: Diabetic retinopathy
ADA: American diabetes association
SIVA: Singapore I Vessel Assessment
ICC: Intra-class correlation coefficient
SBP: Systolic blood pressure
DBP: Diastolic blood pressure
BMI: Body mass index
HDL: High-density lipoprotein
LDL: Low-density lipoprotein
uACR: Urine albumin/creatinine ratio
